# Tumor microenvironment-driven resistance to immunotherapy in non-small cell lung cancer: strategies for Cold-to-Hot tumor transformation

**DOI:** 10.20517/cdr.2025.14

**Published:** 2025-04-24

**Authors:** Jinglu Yu, Xiaoni Kong, Yu Feng

**Affiliations:** ^1^Institute of Integrated Chinese and Western Medicine, PuDong Traditional Chinese Medicine Hospital Affiliated to Shanghai University of Traditional Chinese Medicine, Shanghai 201200, China.; ^2^Institute of Respiratory Medicine, PuDong Traditional Chinese Medicine Hospital Affiliated to Shanghai University of Traditional Chinese Medicine, Shanghai 201200, China.; ^3^Central Laboratory, Shuguang Hospital Affiliated to Shanghai University of Traditional Chinese Medicine, Shanghai 201203, China.

**Keywords:** Tumor microenvironments, non-small cell lung cancer, immunotherapy resistance, Cold-to-Hot tumor transformation, mechanism, strategy

## Abstract

Non-small cell lung cancer (NSCLC) represents a formidable challenge in oncology due to its molecular heterogeneity and the dynamic suppressive nature of its tumor microenvironment (TME). Despite the transformative impact of immune checkpoint inhibitors (ICIs) on cancer therapy, the majority of NSCLC patients experience resistance, necessitating novel approaches to overcome immune evasion. This review highlights shared and subtype-specific mechanisms of immune resistance within the TME, including metabolic reprogramming, immune cell dysfunction, and physical barriers. Beyond well-characterized components such as regulatory T cells, tumor-associated macrophages, and myeloid-derived suppressor cells, emerging players - neutrophil extracellular traps, tertiary lymphoid structures, and exosomal signaling networks - underscore the TME’s complexity and adaptability. A multi-dimensional framework is proposed to transform cold, immune-excluded tumors into hot, immune-reactive ones. Key strategies include enhancing immune infiltration, modulating immunosuppressive networks, and activating dormant immune pathways. Cutting-edge technologies, such as single-cell sequencing, spatial transcriptomics, and nanomedicine, are identified as pivotal tools for decoding TME heterogeneity and personalizing therapeutic interventions. By bridging mechanistic insights with translational innovations, this review advocates for integrative approaches that combine ICIs with metabolic modulators, vascular normalizers, and emerging therapies such as STING agonists and tumor vaccines. The synergistic potential of these strategies is poised to overcome resistance and achieve durable antitumor immunity. Ultimately, this vision underscores the importance of interdisciplinary collaboration and real-time TME profiling in refining precision oncology for NSCLC, offering a blueprint for extending these advances to other malignancies.

## INTRODUCTION

Lung cancer remains the leading cause of cancer-related mortality worldwide, with non-small cell lung cancer (NSCLC) constituting approximately 85% of cases^[1,2]^. Immune checkpoint inhibitors (ICIs) targeting PD-1/PD-L1 have transformed NSCLC treatment, offering prolonged survival and durable responses in a subset of patients^[3-6]^. However, clinical success is constrained by widespread intrinsic or acquired resistance, emphasizing the urgent need to decipher the underlying mechanisms of treatment failure^[7-13]^.

NSCLC comprises distinct molecular and histological subtypes - adenocarcinoma, squamous cell carcinoma (SCC), and large cell carcinoma (LCC) - each shaped by unique tumor microenvironment (TME) characteristics that influence immunotherapy responses^[14-24]^. Adenocarcinomas frequently harbor driver mutations such as EGFR, KRAS, and ALK rearrangements, which modulate immune landscapes. EGFR mutations induce immune-cold TMEs characterized by low TMB and Treg accumulation, whereas KRAS co-mutations generate divergent immune profiles, from suppressive environments (KRAS-STK11) to pro-inflammatory states (KRAS-TP53). Similarly, ALK rearrangements foster immune exclusion through reduced PD-L1 expression and impaired T-cell infiltration^[25-28]^.

SCCs, linked to smoking-induced mutagenesis, exhibit higher TMB and neoantigen loads, theoretically enhancing immunogenicity. However, this benefit is countered by PD-L1 and CTLA-4 overexpression, establishing immunosuppressive feedback loops. Additional alterations in FGFR and DNA damage repair pathways further complicate immune responses^[29-31]^. LCC, though less characterized, demonstrates overlapping TME features with adenocarcinoma or SCC, ranging from immune-cold to immune-inflamed phenotypes^[32]^.

Decoding these subtype-specific immune landscapes is crucial for refining therapeutic interventions. TME-driven resistance stems from a confluence of metabolic reprogramming, cellular exclusion, and immunosuppressive crosstalk, necessitating tailored immunotherapy strategies. Recognizing the interplay between shared immune-suppressive mechanisms and subtype-specific heterogeneity underscores the importance of integrated therapeutic approaches^[33-35]^.

This review dissects immune evasion mechanisms within the TME and presents a multi-dimensional framework to transform cold, immune-excluded tumors into hot, immune-reactive ones. Proposed strategies encompass enhancing immune infiltration, modulating suppressive networks, reactivating dormant immune pathways, and applying emerging technologies. By bridging mechanistic insights with therapeutic innovation, we aim to advance durable antitumor immunity and expand treatment options for NSCLC patients.

## SHARED MECHANISMS IN THE TME

**Figure 1:**
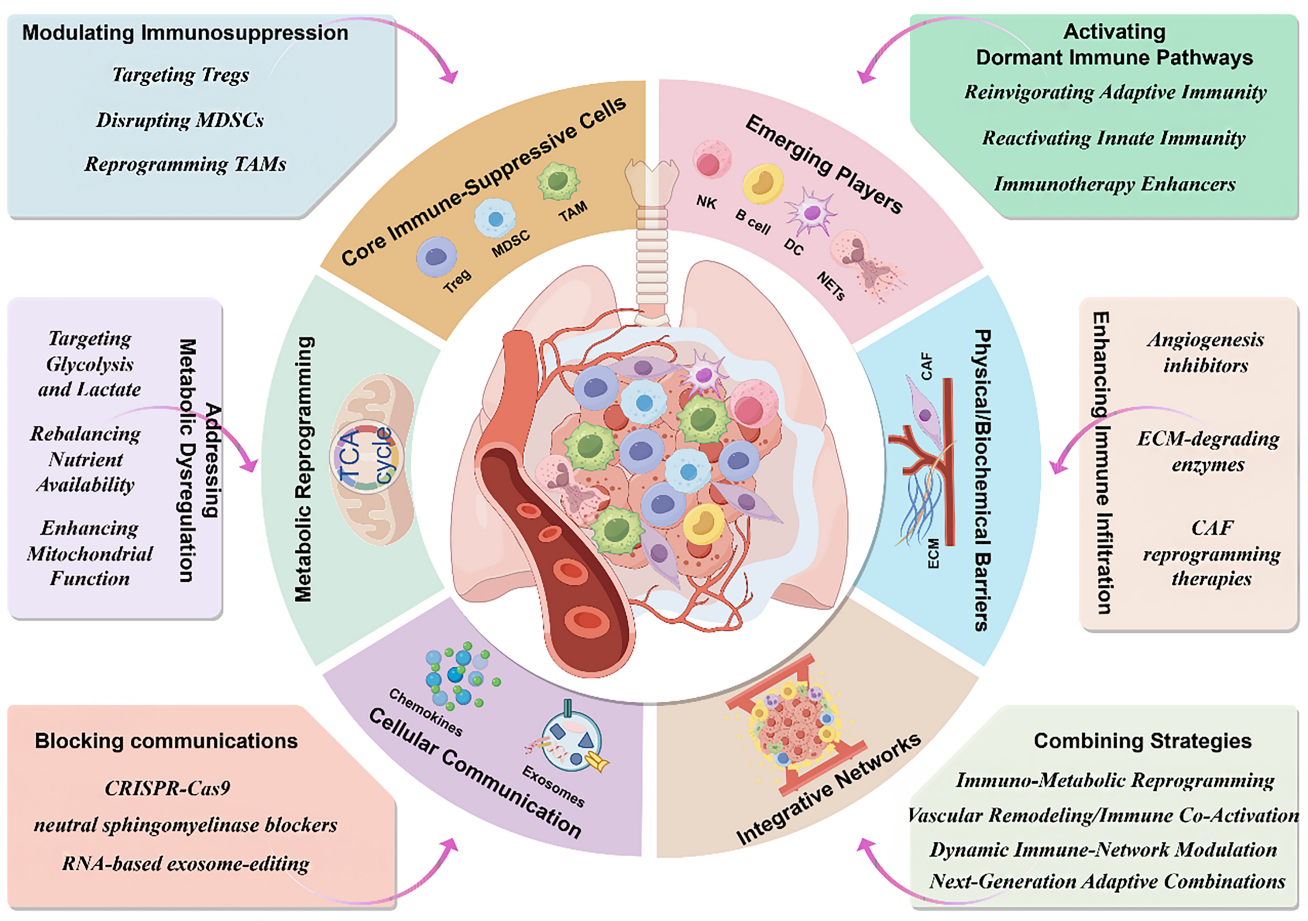
TME-driven strategies for Cold-to-Hot tumor transformation in lung cancer. This figure illustrates a comprehensive framework for reprogramming the TME to overcome immunotherapy resistance in lung cancer, enabling cold-to-hot tumor transformation. The central schematic represents the lung TME, encompassing key components and their interconnections, with surrounding annotations detailing targeted therapeutic strategies. Core immune-suppressive cells, including Tregs, MDSCs, and TAMs, act as critical barriers to effective antitumor immunity. Strategies such as depleting or reprogramming these cells aim to restore immune responsiveness. Emerging players, such as NK cells, B cells, DCs, and NETs, are highlighted as potential contributors to immune activation when properly targeted. The ECM and CAFs are identified as physical and biochemical barriers that impede immune infiltration; therapeutic approaches include ECM-degrading enzymes, angiogenesis inhibitors, and CAF reprogramming therapies. Metabolic reprogramming focuses on addressing tumor-induced metabolic dysregulation, including glycolysis, lactate accumulation, and nutrient imbalances, through interventions that enhance mitochondrial function and restore metabolic homeostasis. Cellular communication within the TME, mediated by chemokines and exosomes, reinforces immunosuppressive networks; strategies such as CRISPR-Cas9, neutral sphingomyelinase blockers, and RNA-based exosome editing hold promise for disrupting these interactions. The integrative networks within the TME underscore the dynamic interplay of immune, metabolic, and structural components, necessitating combined approaches. Highlighted therapeutic strategies include immuno-metabolic reprogramming, vascular remodeling, immune co-activation, and next-generation adaptive combinations. This figure provides a unified view of the TME’s complexity and offers insights into multi-modal strategies to reprogram the TME for improved immunotherapy outcomes. TME: Tumor microenvironment; Tregs: regulatory T cells; MDSCs: myeloid-derived suppressor cells; TAMs: tumor-associated macrophages; NK cells: natural killer cells; DCs: dendritic cells; NETs: neutrophil extracellular traps; ECM: extracellular matrix; CAFs: cancer-associated fibroblasts; CRISPR-Cas9: clustered regularly interspaced short palindromic repeats-associated protein 9; TCA: tricarboxylic acid.

The TME in NSCLC serves as a dynamic ecosystem where multiple suppressive mechanisms converge, fostering immune evasion and therapeutic resistance. Despite subtype-specific variations, common processes such as immune cell dysfunction, metabolic reprogramming, and structural barriers collectively drive tumor progression. These interconnected pathways underscore the TME’s critical role in shaping immune suppression, laying the groundwork for therapeutic interventions^[33,35,36]^ [Figure 1 and Table 1].

**Table 1:** TME-driven mechanisms of immunotherapy resistance and cold tumor characteristics

**Category**		**Mechanism/Characteristic**	**Clinical implication**	**References**
Immunosuppressive cells	Treg	Tregs secrete IL-10 and TGF-β, suppressing effector T cells	Inhibits antitumor immune response	[37,46,47]
	MDSC	MDSCs deplete arginine and tryptophan via ARG1 and IDO, disrupting T cell metabolism, while ROS production impairs TCR signaling	Promotes metabolic exhaustion of T cells and increases oxidative stress, weakening antitumor immunity.	[48]
	TAM	M2-polarized TAMs secrete VEGF and remodel ECM, forming barriers to immune infiltration	Promotes vascular abnormalities and immune exclusion, creating a pro-tumor niche resistant to immunotherapy	[49,50]
Emerging players	NETs	Neutrophils form NETs via NETosis, releasing DNA and enzymes that shield tumor cells and suppress immune surveillance	Induces CD8+ T cell exhaustion and supports metastases via A2AR-CCL5 and NF-κB/NLRP3 pathways	[51-53]
	TLSs	TLSs, composed of B cell follicles, T cell zones, and HEVs, recruit and activate immune cells through CXCL13 and CCL19 signaling	Organized TLSs enhance antigen presentation and boost ICI responses, while disorganized TLSs attract Tregs, promoting immune evasion	[54-56]
	B cells	B cells and plasma cells support TLS formation and enhance immune surveillance via antigen presentation	Strengthen responses to immune checkpoint blockade in NSCLC	[55,57,58]
	Complement system	The complement system promotes immunosuppression through elevated C5a levels, driven by circASCC3-mediated miR-432-5p sponging	Drives immune evasion and metastasis, correlating with poor immunotherapy outcomes	[59]
	Tex	Tex suppress immune responses by losing cytotoxic functions and secreting immunosuppressive cytokines, while maintaining persistent expression of inhibitory receptors like PD-1, TIM-3, and LAG-3	A progenitor-like Tex subset, regulated by TCF-1, retains proliferative potential and responsiveness to ICIs	[60-63]
	NK cells	NK cells lose cytotoxicity in the tumor microenvironment due to TGF-β and lactate, which downregulate activating receptors (e.g., NKG2D) and upregulate inhibitory receptors (e.g., KIR)	Restoring NK cell function through IL-15, bispecific NK cell engagers, and lactate-targeted metabolic interventions offers new therapeutic avenues	[65,66]
Metabolic reprogramming	Lactate	Lactate accumulation acidifies the TME, suppresses CTL and NK cell activity, and induces M2 macrophage polarization by stabilizing HIF-1α	Acidification limits immune cell infiltration and activity, while lactate-driven VEGF and IL-10 secretion reinforces tumor growth and immune evasion	[66,68,69,70,71,72]
	Nutrient depletion	Nutrient deprivation induces M2 macrophage polarization through arginine and tryptophan depletion, stabilizing HIF-1α and enhancing VEGF and IL-10 secretion	Limits immune effector cell function, reduces the response to ICIs, and enhances resistance	[67,73,74,75,76,77,78]
	Fatty acid	Altered fatty acid metabolism drives immune dysfunction by promoting fatty acid oxidation (FAO) in tumor cells and lipid accumulation in dendritic cells (DCs), disrupting CTL function and antigen presentation	Fatty acid metabolism reshapes the TME to impair CTL-mediated cytotoxicity and adaptive immunity, reducing the effectiveness of ICIs and supporting tumor progression	[79-82]
Physical and biochemical barriers	ECM	ECM remodeling by CAFs generates a dense fibrotic matrix, excluding CTLs and NK cells and disrupting immune cell migration through increased tissue stiffness	ECM remodeling promotes immune exclusion, impairs T cell and NK cell-mediated tumor killing, and reduces responsiveness to ICIs	[83,84]
	Angiogenesis	Abnormal angiogenesis driven by VEGF produces structurally defective, leaky vessels, hindering immune cell trafficking and creating a hypoxic, nutrient-deprived niche	Impaired vascular integrity promotes immune exclusion, MDSC recruitment, and TAM polarization, reducing ICI efficacy	[85-87]
	Chemokine	Chemokine dysregulation shifts the balance toward immunosuppressive signals (e.g., CCL2, CXCL12), recruiting MDSCs, TAMs, and Tregs while reducing effector chemokines (CXCL9, CXCL10) needed for CTL infiltration	Chemokine imbalances create immune-cold tumor regions, hindering CTL recruitment and reducing ICI efficacy, contributing to immune exclusion	[88]
Cellular communication	Cytokine and chemokine networks	Cytokine and chemokine networks amplify immunosuppression by sustaining CAF, TAM, and Treg activity through IL-6 and TGF-β signaling while impairing CTL and NK cell functions	Reinforces immune suppression locally and systemically, reducing ICI efficacy and facilitating tumor immune evasion	[89-91]
	EVs	EVs, particularly tumor-derived exosomes, transport PD-L1 and TGF-β to inhibit TCR activation, disrupt antigen presentation, and prime pre-metastatic niches	EV-mediated immune suppression facilitates long-range immune evasion, reduces ICI efficacy, and promotes metastatic progression	[92-95]
	Signaling pathways	Signaling pathways like cGAS-STING and RIG-I/MAVS regulate immune activation through IFN-I production, balancing pro- and antitumor immune responses. Chronic cGAS-STING activation recruits MDSCs, while RIG-I/MAVS enhances CTL function and antitumor immunity	Dysregulated signaling pathways contribute to immune suppression and tumor progression, presenting opportunities to reprogram the TME for enhanced ICI efficacy	[96-102]
Integrative networks	Feedback loops	Hypoxia-induced glycolysis elevates lactate production, stabilizing HIF-1α and promoting TAM polarization and VEGF-mediated angiogenesis, while CAF-driven ECM remodeling disrupts vascular integrity, worsening hypoxia and nutrient deprivation	Feedback loops between metabolic reprogramming and ECM remodeling create immune-excluded zones, impairing ICI efficacy and promoting tumor progression. Targeting these loops through ECM normalization and metabolic interventions offers potential therapeutic strategies	[103-106]
	Systemic coordination	Tumor-derived exosomes carrying PD-L1 and IDO suppress TCR signaling and deplete nutrients, while the cGAS-STING pathway bridges local and systemic immunity through type I interferon responses and chronic MDSC recruitment	Systemic immunosuppression impairs ICI efficacy and promotes tumor progression. Targeting both local and systemic drivers offers the potential for reversing immune escape	[96,97,98,99,107,108]
	Adaptive resistance	Adaptive resistance in the TME emerges through compensatory mechanisms, such as VEGF inhibition-induced TAM and MDSC recruitment or lactate targeting triggering oxidative stress, impairing CTL function	Single-target therapies often fail due to dynamic adaptations in the TME. Multi-modal strategies targeting interconnected suppressive networks are essential to sustain immune activation and improve ICI efficacy	[109-114]

Treg: Regulatory T Cells; IL-10: interleukin 10; TGF-β: transforming growth factor beta; MDSC: myeloid-derived suppressor cell; ARG1: arginase 1; IDO: indoleamine 2,3-dioxygenase; ROS: reactive oxygen species; TCR: T cell receptor; TAM: tumor-associated macrophage; VEGF: vascular endothelial growth factor; ECM: extracellular matrix; NETs: neutrophil extracellular traps; CD8: cluster of differentiation 8; A2AR: adenosine 2A receptor; CCL5: C-C motif chemokine ligand 5; NF-κB: nuclear factor kappa-light-chain-enhancer of activated B Cells; NLRP3: NOD-, LRR-, and pyrin domain-containing protein 3; TLSs: tertiary lymphoid structures; HEVs: high endothelial venules; CXCL13: C-X-C motif chemokine ligand 13; CCL19: C-C motif chemokine ligand 19; ICI: immune checkpoint inhibitor; NSCLC: non-small cell lung cancer; C5a: complement component 5a; circASCC3: circular RNA ASCC3; miR-432-5p: MicroRNA 432-5p; Tex: exhausted T cells; PD-1: programmed death-1; TIM-3: T cell immunoglobulin and mucin domain-containing protein 3; LAG-3: lymphocyte activation gene-3; TCF-1: T cell factor 1; NK cells: natural killer cells; NKG2D: natural killer group 2 member D; KIR: killer cell immunoglobulin-like receptor; CTL: cytotoxic T lymphocyte; HIF-1α: hypoxia-inducible factor 1 alpha; CAFs: cancer-associated fibroblasts; CCL2: C-C motif chemokine ligand 2; CXCL12: C-X-C motif chemokine ligand 12; CXCL9: C-X-C motif chemokine ligand 9; CXCL10: C-X-C motif chemokine ligand 10; IL-6: interleukin 6; cGAS: cyclic GMP-AMP synthase; STING: stimulator of interferon genes; RIG-I: retinoic acid-inducible gene I; MAVS: mitochondrial antiviral signaling protein; IFN-I: type I interferon; PD-L1: programmed death-ligand 1; Evs: extracellular vesicles.

### Core immune-suppressive cells in the TME

Key immunosuppressive cells - including regulatory T cells (Tregs), myeloid-derived suppressor cells (MDSCs), and tumor-associated macrophages (TAMs) - form the immunosuppressive core of the NSCLC TME by suppressing cytotoxic T lymphocytes (CTLs), dampening antigen presentation, and promoting tumor progression^[37-45]^.

Tregs are actively recruited through tumor-derived chemokines such as CCL22 and CXCL17. Upon infiltration, they secrete suppressive cytokines, including transforming growth factor-beta (TGF-β) and IL-10, which inhibit CTL proliferation and impair dendritic cells (DCs)-mediated antigen presentation^[37]^. This mechanism is particularly pronounced in KRAS-mutant adenocarcinomas, where MEK-ERK-AP1 signaling fosters Treg recruitment, exacerbating immune resistance^[46,47]^.

MDSCs, mobilized by tumor-secreted factors such as GM-CSF, VEGF, and IL-6, suppress immune responses through multiple pathways. They deplete essential metabolites like arginine and tryptophan through enzymes such as arginase 1 (ARG1) and indoleamine 2,3-dioxygenase (IDO), respectively. Additionally, they produce reactive oxygen species (ROS), which impair T cell receptor (TCR) signaling and increase oxidative stress, correlating with ICI resistance^[48]^.

TAMs are the most abundant immune cells in the TME, predominantly adopting an M2-like immunosuppressive phenotype driven by hypoxia and cytokines such as IL-10 and TGF-β^[49]^. These M2-polarized TAMs promote angiogenesis via VEGF secretion, remodel the extracellular matrix (ECM), and create physical barriers that block immune cell infiltration. Hypoxia stabilizes HIF-1α, reinforcing TAM-mediated suppression through a self-sustaining feedback loop^[50]^.

The synergistic interactions among Tregs, MDSCs, and TAMs reinforce an immunosuppressive environment, limiting the efficacy of ICIs. Understanding these cellular dynamics provides a foundation for developing targeted strategies to reinvigorate antitumor immunity.

### Emerging players in the TME

Beyond well-characterized suppressive components such as Tregs, MDSCs, and TAMs, several emerging players in the TME further complicate immune resistance in NSCLC. These include neutrophil extracellular traps (NETs), tertiary lymphoid structures (TLSs), B cells, exhausted T cells (Tex), and natural killer (NK) cells. Their integration into established immunosuppressive networks underscores the need for multi-targeted therapeutic approaches.

#### NETs and NETosis: immune suppression and structural barriers

Neutrophils contribute to immune suppression through NETosis, releasing DNA, histones, and enzymes that form NETs. These web-like structures shield tumor cells from immune surveillance while fostering a pro-inflammatory but immunosuppressive environment^[51]^. NETs promote CD8+ T cell exhaustion by activating A2AR-CCL5 signaling^[52]^, driving both local and distant metastases in an NF-κB/NLRP3-dependent manner in NSCLC^[53]^. Their association with poor clinical outcomes highlights NETs as potential therapeutic targets.

#### TLSs: immune hubs or immunosuppressive niches

TLSs are ectopic lymphoid formations composed of B cell follicles, T cell zones, and high endothelial venules (HEVs), facilitating immune cell recruitment and activation. Well-organized TLSs enhance local antigen presentation, boosting antitumor immunity and correlating with improved responses to ICIs. However, disorganized TLSs can attract suppressive immune cells such as Tregs, contributing to immune evasion. Key regulators such as CXCL13 and CCL19 modulate TLS functionality, offering therapeutic targets for TLS-driven immunomodulation^[54-56]^.

#### B cells and the complement system

Recently, B cells and plasma cells, once considered minor contributors to antitumor immunity, have emerged as key players in checkpoint blockade responses in NSCLC patients^[57,58]^. They contribute to TLS formation, promoting immune surveillance through antigen presentation^[55]^. Conversely, the complement system, through elevated C5a levels driven by circASCC3-mediated miR-432-5p sponging, promotes NSCLC progression and fosters an immunosuppressive TME, underscoring its role in shaping resistance to NSCLC immunotherapy^[59]^. Their context-dependent roles highlight the complexity of targeting TME components in NSCLC.

#### Tex: reversible dysfunction

Chronic antigen stimulation, coupled with suppressive signals from Tregs, hypoxia, and metabolic stress, drives T cell exhaustion, characterized by the loss of effector functions and persistent expression of inhibitory receptors such as PD-1, TIM-3, and LAG-3^[60,61]^. Recent research highlights a progenitor-like Tex subset with proliferative potential and responsiveness to ICIs in NSCLC, regulated by transcription factors such as TCF-1^[62,63]^. Unlocking this potential through checkpoint inhibitors and epigenetic modulators remains a promising therapeutic strategy.

#### NK cells: restoring innate immunity

NK cells, essential for innate immunity, are often suppressed due to tumor-microenvironment-derived factors such as TGF-β and lactate. These factors downregulate activating receptors (e.g., NKG2D) while upregulating inhibitory receptors (e.g., KIR), impairing NK cell-mediated cytotoxicity^[64]^. Therapeutic strategies to restore NK cell function include cytokine-based therapies such as IL-15, bispecific NK cell engagers, and metabolic interventions targeting lactate accumulation^[65,66]^.

Emerging players such as NETs, TLSs, B cells, Tex, and NK cells integrate into established immunosuppressive networks, reinforcing immune evasion through complementary and overlapping mechanisms. Their deep interconnection with traditional components underscores the necessity of multi-pronged therapeutic strategies targeting the full complexity of the TME.

### Metabolic reprogramming: a hostile microenvironment

Metabolic reprogramming within the TME establishes a hostile, nutrient-deprived niche that promotes immune evasion and tumor progression. Tumor cells exploit metabolic shifts to meet their energy demands, outcompete immune cells, and create a suppressive environment^[67]^.

#### Lactate accumulation: a barrier to immune activation

Driven by aerobic glycolysis (the Warburg effect), tumor cells produce excessive lactate, acidifying the TME and impairing CTLs and NK cells^[66,68-70]^. Lactate induces M2 polarization in TAMs, enhancing their secretion of VEGF and IL-10 while stabilizing HIF-1α to sustain angiogenesis and hypoxia^[71,72]^. This forms a self-reinforcing feedback loop that fortifies immune exclusion.

#### Nutrient depletion: immune starvation

Resource competition exacerbates immune suppression as tumor cells outcompete effector T cells for glucose, impairing their cytokine production and proliferation^[67]^. Enzymes like ARG1 and IDO deplete essential amino acids such as arginine and tryptophan, further restricting T cell activation^[73]^ while fostering Treg and MDSC expansion^[74-76]^. Glutamine metabolism fuels anabolic processes in tumor cells, deepening resource depletion and further crippling immune responses^[77,78]^.

#### Fatty acid metabolism and immune dysfunction

Altered fatty acid metabolism adds another dimension of immune dysfunction. Tumor cells rely on fatty acid oxidation (FAO) to produce lipid mediators that suppress CTL function^[79,80]^. Simultaneously, lipid accumulation in DCs disrupts antigen presentation, weakening adaptive immunity^[81,82]^. These interlinked metabolic alterations collectively promote tumor progression and immune resistance.

Metabolic reprogramming drives an interconnected web of immune evasion, where tumor-promoting pathways create a deeply suppressive ecosystem. Targeting these processes holds significant potential for therapeutic intervention in NSCLC.

### Physical and biochemical barriers: excluding effector cells

Physical and biochemical barriers in the TME synergistically limit immune cell infiltration and effector function. These barriers form a dynamic network closely integrated with metabolic and cellular processes, reinforcing immune evasion and therapeutic resistance.

#### ECM remodeling

Cancer-associated fibroblasts (CAFs) play a central role in reshaping the ECM, generating a fibrotic scaffold that impedes immune cell infiltration. By producing dense matrix proteins such as collagen and fibronectin, CAFs create a structural barrier excluding CTLs and NK cells from the tumor core. Tissue stiffness caused by ECM remodeling further disrupts immune cell migration. In parallel, CAF-derived cytokines, notably, promote immune suppression by enhancing Treg recruitment and limiting effector cell activity^[83,84]^.

#### Abnormal angiogenesis

Tumor-induced angiogenesis results in structurally defective, poorly perfused blood vessels characterized by excessive branching and leakiness. These abnormal vessels hinder immune cell trafficking and nutrient delivery, creating a hypoxic and nutrient-deprived niche. Vascular endothelial growth factor (VEGF), a key angiogenic driver, selectively recruits MDSCs while excluding CTLs^[85,86]^. Hypoxia-induced stabilization of hypoxia-inducible factor 1-alpha (HIF-1α) exacerbates immunosuppression by polarizing TAMs toward the pro-tumorigenic M2 phenotype^[87]^.

#### Chemokine dysregulation

Chemokine imbalances further intensify immune exclusion in the TME. Tumor and stromal cells secrete immunosuppressive chemokines such as CCL2 and CXCL12, which attract MDSCs, TAMs, and Tregs while downregulating effector chemokines like CXCL9 and CXCL10 that recruit CTLs. This selective chemokine gradient creates spatially segregated immune suppression, particularly in immune-cold tumor regions resistant to ICIs^[88]^.

By interweaving these structural and biochemical barriers, the TME constructs a formidable immune-evasive network, limiting therapeutic efficacy and underscoring the need for multifaceted strategies targeting these barriers.

### Cellular communication: expanding immune regulation networks

Tumor progression and immune evasion in NSCLC are not driven by isolated molecular events but by an intricate network of cellular communication. This includes the integration of cytokines, extracellular vesicles (EVs), and intracellular signaling pathways, which collectively amplify immune suppression and sustain therapeutic resistance.

#### Cytokine and chemokine networks: amplifying immunosuppression

Building on chemokine-driven immune exclusion, cytokines further expand immunosuppressive networks within the TME^[88]^. Pro-inflammatory mediators such as IL-6 and TGF-β establish a feedback loop that sustains CAF, TAM and Treg activity while impairing CTL and NK cell functions^[89-91]^. These cytokines not only reinforce local immune suppression but also enable systemic immunosuppressive signaling, promoting tumor persistence despite immune surveillance. Their dynamic interplay with chemokines underscores the tightly orchestrated immune-modulating circuits that define the TME.

#### EVs: mediators of long-range immune evasion

EVs, particularly tumor-derived exosomes (TDEs), extend immunosuppressive signaling beyond the local TME^[92]^. By transporting PD-L1 and TGF-β, EVs inhibit TCR activation, disrupt antigen presentation, and establish pre-metastatic niches in distant tissues^[93-95]^. This vesicle-mediated crosstalk not only sustains immune evasion but also primes sites for metastatic colonization, presenting a promising target for therapeutic disruption.

#### Signaling pathways: orchestrating immune escape and tumor progression

Central to immune regulation are signaling pathways that modulate immune activation and suppression. The cGAS-STING pathway, for instance, mediates innate immune activation through IFN-I production upon detecting cytosolic DNA^[96,97]^. However, chronic activation of this pathway paradoxically recruits MDSCs and triggers immune suppression^[98,99]^. Similarly, RIG-I/MAVS signaling, while detecting cytosolic RNA and activating IFN-I responses^[100]^, contributes to enhancing antitumor immunity, thereby suppressing tumor progression^[101,102]^. Balancing these pathways could unlock new therapeutic opportunities by shifting the TME toward an immune-reactive state.

The TME constructs a local-to-systemic immune evasion network through cytokines, exosomes, and signaling pathways, dynamically regulating immunosuppression and tumor progression while offering critical targets for multi-level therapeutic interventions.

### Integrative networks in the TME

The TME operates as a dynamic and interdependent system where immune-suppressive mechanisms integrate through metabolic, structural, and cellular communication networks. These feedback loops collectively sustain an immunosuppressive milieu resistant to therapeutic intervention, necessitating a multi-targeted therapeutic approach.

#### Feedback loops between metabolic and structural barriers

Metabolic reprogramming and ECM remodeling form a tightly interconnected suppressive axis within the TME. Hypoxia-induced glycolysis elevates lactate production, which acidifies the TME and stabilizes HIF-1α, promoting TAM polarization and VEGF-mediated angiogenesis^[103,104]^. Simultaneously, CAF-driven ECM remodeling disrupts vascular integrity, worsening hypoxia and reinforcing nutrient deprivation^[105,106]^. This dual mechanism generates physical and metabolic barriers that hinder immune cell infiltration and function, creating immune-excluded zones. Disrupting these feedback loops through ECM normalization and metabolic targeting holds promise for reversing immune suppression.

#### Systemic coordination through cellular communication

Beyond localized interactions, the TME exerts systemic immune modulation via EVs and immunomodulatory signaling. TDEs transport PD-L1 and IDO, amplifying immune suppression by suppressing TCR signaling and depleting critical nutrients^[107,108]^. Similarly, the cGAS-STING pathway bridges local and systemic immunity by initiating type I interferon responses but can paradoxically sustain immunosuppression through chronic MDSC recruitment and inflammation^[96-99]^. These mechanisms emphasize the need to target both local and systemic immunosuppressive drivers.

#### The challenge of adaptive resistance

The TME’s resilience is driven by its adaptive capacity. For instance, VEGF inhibition may transiently normalize vasculature but induce compensatory recruitment of suppressive TAMs and MDSCs^[109-112]^. Similarly, targeting metabolic vulnerabilities like lactate production risks triggering oxidative stress, further impairing CTL activity^[113,114]^. These dynamic adaptations highlight the limitations of single-target therapies, underscoring the importance of multi-modal strategies that disrupt interdependent suppressive networks and sustain immune activation.

## HETEROGENEITY IN THE TME

**Figure 2:**
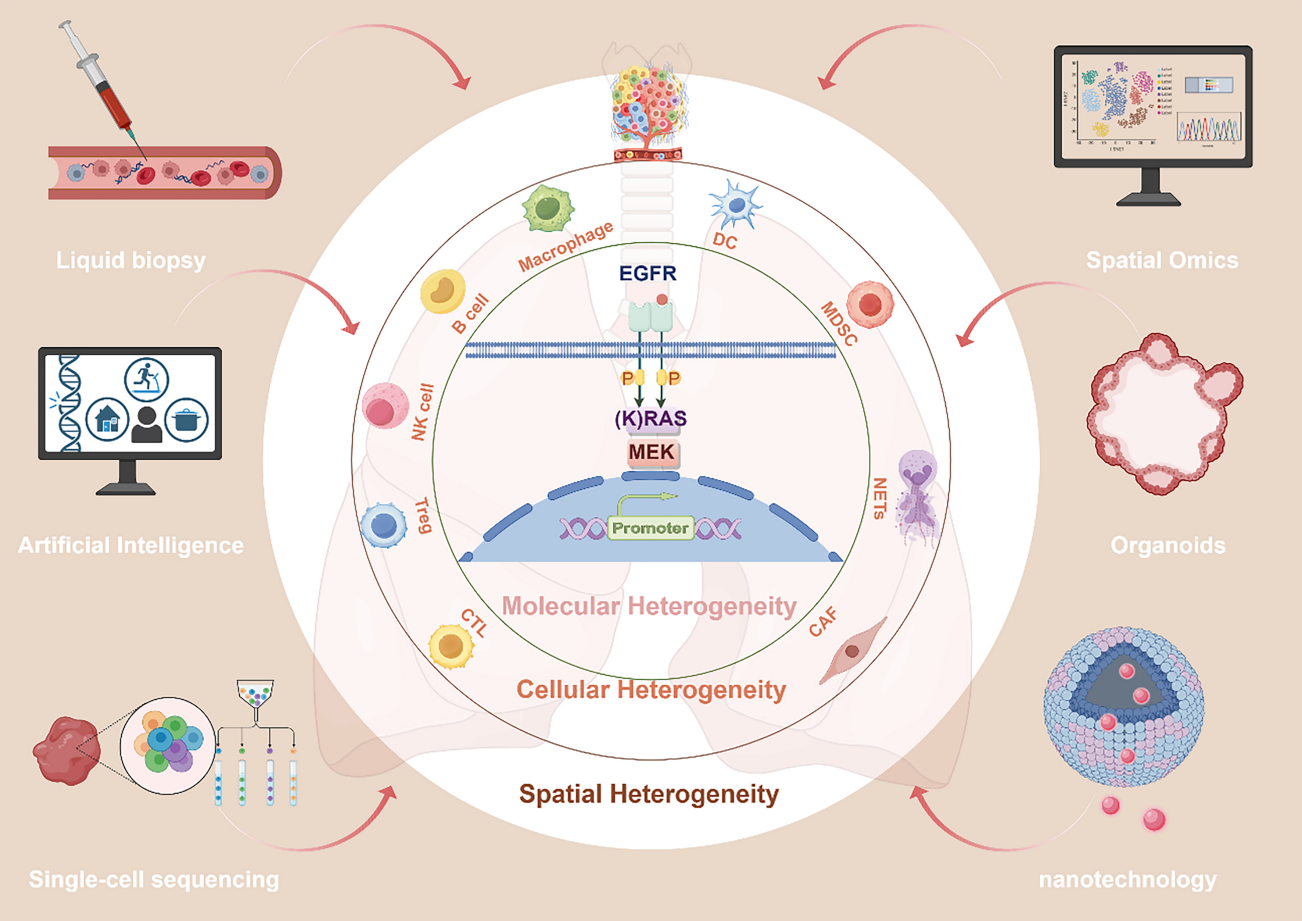
Integrative analysis of tumor heterogeneity and emerging technologies in lung cancer immunotherapy. This concentric illustration highlights the multi-dimensional heterogeneity of the TME, emphasizing molecular, cellular, and spatial complexity in lung cancer. Molecular heterogeneity, driven by genetic mutations (e.g., EGFR, KRAS) and epigenetic alterations activating oncogenic pathways such as RAS-MEK signaling, forms the core of tumor progression and immune evasion. Cellular heterogeneity emerges through complex interactions among immune and stromal cell subsets - including Tregs, NK cells, CTLs, MDSCs, TAMs, CAFs, and NETs - facilitating immunosuppression. At the outer layer, spatial heterogeneity reflects cell organization into immune-active hotspots and immunologically cold zones shaped by physical barriers, such as the ECM. Advanced technologies, including spatial omics, single-cell sequencing, liquid biopsy, organoids, AI analytics, and nanotechnology, enable precise characterization and therapeutic targeting of these heterogeneities. TME: Tumor microenvironment; EGFR: epidermal growth factor receptor; KRAS: Kirsten rat sarcoma viral oncogene homolog; RAS-MEK: RAS- mitogen-activated protein kinase kinase; Tregs: regulatory T cells; NK cells: natural killer cells; CTLs: cytotoxic T lymphocytes; MDSCs: myeloid-derived suppressor cells; TAMs: tumor-associated macrophages; CAFs: cancer-associated fibroblasts; ECM: extracellular matrix; NETs: neutrophil extracellular traps.

The TME operates as a dynamic and adaptive system driven by interlinked suppressive networks involving metabolic shifts, cellular communication, and structural remodeling. However, the degree to which these mechanisms manifest is far from uniform. TME exhibit striking heterogeneity shaped by distinct tumor subtypes, mutational landscapes, and external pressures from therapeutic interventions. This spatial and molecular diversity creates unique immune landscapes within different lung cancer subtypes, posing a significant challenge for developing universal therapeutic strategies. Understanding this heterogeneity is essential for tailoring precision therapies that target the specific vulnerabilities of each immune-suppressive niche^[33-35]^ [Figure 2].

### Molecular heterogeneity: genetic and epigenetic drivers

Molecular heterogeneity arises from genetic mutations, epigenetic modifications, and transcriptomic variability, creating distinct immunological profiles within NSCLC subtypes. EGFR, KRAS, and ALK alterations influence TMB, neoantigen presentation, and immune evasion. Epigenetic modifiers, such as histone deacetylases, further regulate immune gene expression, shaping the suppressive molecular milieu. Comprehensive molecular profiling enables subtype-specific therapeutic approaches, including combining ICIs with targeted inhibitors or epigenetic reprogrammers^[12-15,19-23]^.

### Cellular heterogeneity: diverse immunosuppressive ecosystems

The cellular landscape of the TME is defined by a dynamic balance between immune effector and suppressor populations. While the presence of Tregs, MDSCs, and TAMs is well-characterized, emerging players like neutrophils, B cells, and progenitor-like Tex contribute additional complexity. In NSCLC, distinct immune cell compositions correlate with specific molecular subtypes. For instance, EGFR-mutant adenocarcinomas exhibit elevated Tregs and low effector T cell infiltration, while SCCs display robust but suppressed immune infiltrates due to extensive immune checkpoint upregulation^[22,26,28-30,33,37,48,51,55]^. The balance among these components shapes the immune contexture and determines the therapeutic potential of immunotherapy.

### Spatial heterogeneity: immune zoning and functional niches

Spatial compartmentalization within the TME creates immune-exclusive zones that limit the effectiveness of immunotherapy. Tumor cores often exhibit dense ECM barriers, hypoxic niches, and metabolic deprivation, forming “cold” regions resistant to immune infiltration. Conversely, peritumoral regions may host active TLSs or effector T cell clusters, contributing to localized immune activation. Techniques such as spatial transcriptomics and multiplex imaging have revealed this heterogeneity, emphasizing the need for therapies targeting both immune-excluded and inflamed regions simultaneously^[36,58,115-120]^.

### Temporal heterogeneity: dynamic evolution of immune contexture

Tumor heterogeneity in NSCLC is a dynamic process that evolves across disease progression and under therapeutic pressure, known as temporal heterogeneity. Early immune-evasive mechanisms - marked by increased immune checkpoint expression (PD-L1, CTLA-4, IDO1) - occur even in preinvasive stages, accompanied by structural segregation of immune and epithelial cells, highlighting early temporal remodeling of the TME^[121]^. As lung adenocarcinoma progresses from preinvasive to invasive stages, genomic subclonal mutations accumulate, closely paralleling changes in immune infiltration patterns, including significantly increased infiltration of CD8^+^ T cells^[122]^. Single-cell analyses further reveal ongoing temporal evolution within tumor-infiltrating T cell populations, emphasizing continuous immune adaptation during tumor progression^[123]^. Importantly, therapeutic interventions, particularly anti-PD-1 therapies, profoundly reshape the tumor immune landscape by driving substantial infiltration of novel T cell clones, a process termed "clonal replacement," rather than merely reactivating existing *Tex*^[124]^. These findings collectively underscore temporal heterogeneity as a critical factor influencing treatment responses, advocating longitudinal TME monitoring through serial biopsies and spatial omics to optimize personalized NSCLC therapy.

The profound heterogeneity within the TME highlights the need for multifaceted therapeutic approaches. A single-target strategy is insufficient against such a complex, evolving ecosystem. Exploring innovative strategies to transform cold, immune-excluded tumors into hot, immune-responsive environments by leveraging new technologies and combination therapies is imperative for maximizing therapeutic potential.

## STRATEGIES FOR COLD-TO-HOT TUMOR TRANSFORMATION

Transforming immune-cold tumors into immune-reactive or “hot” tumors is a central challenge in overcoming immunotherapy resistance in NSCLC^[116-120]^. Achieving this requires targeting the interconnected mechanisms of immune suppression, cellular exclusion, and metabolic reprogramming within TME^[33-35]^. Building on the insights into shared mechanisms and subtype-specific heterogeneity, strategies to enhance immune infiltration, counteract suppressive cells, reactivate dormant immune pathways, and modulate metabolic constraints form the cornerstone of this transformation. By integrating these approaches with advanced technologies and combination therapies, it becomes possible to dismantle cold tumor barriers and unlock the full potential of immunotherapy, enabling sustained and effective antitumor responses [Figure 1 and Table 2].

**Table 2:** Strategies for cold tumor transformation and overcoming resistance

**Combination strategy**	**Underlying mechanism**	**Preclinical/Clinical evidence**	**Challenges and future directions**	**References**
VEGF inhibitors	Normalizes vasculature, improving oxygenation and T cell infiltration	Atezolizumab + bevacizumab showed clinical benefits (IMbrave150)	Requires optimized dosing and biomarker-based patient selection	[125-131]
ECM-targeting approaches	Disrupts fibrotic barriers and reprograms CAFs to support immune infiltration	Antifibrotic agents (e.g., losartan) and ECM-degrading enzymes (e.g., PEGPH20) enhanced CTL migration in preclinical studies	Long-term safety and combination strategies need validation	[132-139]
Chemokine modulation	Rebalances chemokine gradients to enhance CTL recruitment and limit MDSC/TAM attraction	CCR2 inhibitors block CCL2, and engineered DCs expressing CXCL9/10 restore CTL attraction in preclinical studies	Requires validation of long-term efficacy and safety of gene-editing technologies (e.g., CRISPR)	[140-146]
Treg-targeting therapies	Depletes Tregs in the TME while sparing effector T cells using CCR4 antagonists, anti-CD25 agents, and anti-CTLA-4 variants	Anti-CTLA-4 variants with modified Fc domains show preclinical success in enhancing antitumor immunity	Requires balancing Treg depletion with maintaining immune tolerance to avoid autoimmunity	[147-149]
MDSC-targeting therapies	Inhibit MDSC-mediated suppression via arginase inhibitors (e.g., INCB001158), IDO inhibitors (e.g., epacadostat), and CXCR2 blockade	INCB001158 + pembrolizumab demonstrated enhanced antitumor activity in phase I/II trials for solid tumors	Requires integration of real-time MDSC profiling to optimize timing and combination strategies	[150-152]
TAM reprogramming	Converts TAMs from M2-like (immunosuppressive) to M1-like (pro-inflammatory) states using CSF-1R inhibitors, PI3Kγ inhibitors, and synthetic TLR or STING agonists	CSF-1R inhibitor LY3022855 and PI3Kγ inhibitors demonstrated enhanced M1 polarization and T cell priming in phase I trials	Optimizing combination with ICIs and addressing potential off-target effects	[153-156]
TDE-targeting therapies	Disrupt TDE secretion via neutral sphingomyelinase inhibitors (e.g., GW4869) or neutralize TDE cargo (e.g., PD-L1) using engineered nanobodies	Nanobodies neutralizing exosomal PD-L1 reversed T cell exhaustion in preclinical models	Translating exosome-editing therapies into clinical applications requires addressing delivery and specificity challenges	[157-159]
TCR signaling modulation	Restores TCR function through dual checkpoint blockade (e.g., PD-1/LAG-3) and engineered T cell therapies, including CAR-T cells with advanced costimulatory domains	PD-1/LAG-3 blockade demonstrated improved T cell persistence in clinical trials; CAR-T cells integrating ICOS or 4-1BB costimulation enhanced antitumor responses in preclinical models	Balancing specificity and off-target risks in CAR-T design; optimizing scalability of allogeneic CAR-T platforms	[160-168]
Memory T cell enhancement	Promotes memory T cell differentiation and survival via IL-7/IL-15 superagonists, mitochondrial health regulation (PGC-1α), and mRNA vaccines targeting neoantigens	IL-7/IL-15 agonists and neoantigen mRNA vaccines have shown clinical efficacy in enhancing long-term tumor control	Optimizing personalized vaccine platforms and addressing variability in T cell memory formation	[169-176]
NK cell restoration	Enhances NK cell cytotoxicity via metabolic reprogramming (e.g., AMPK agonists) and precise targeting with bispecific NK engagers (e.g., AFM24)	CAR-NKs and bispecific NK engagers (e.g., AFM24) have shown superior persistence and tumor targeting in clinical trials	Overcoming TME resistance and optimizing NK cell delivery systems for broad clinical application	[177-180]
DC activation strategies	Enhances antigen cross-presentation and adaptive immunity through TLR agonists (e.g., TLR7/8), STING agonists (e.g., ADU-S100), and DC vaccines	DC vaccines loaded with tumor-specific peptides demonstrated improved T cell responses in checkpoint inhibitor-resistant NSCLC patients	Optimizing delivery systems (e.g., biomaterial scaffolds) and ensuring long-term safety of adjuvant therapies	[181-187]
Immune pathway activation	Reprograms suppressive TME through RIG-I agonists (e.g., FLT3L analogs) and cGAS-STING agonists (e.g., ADU-S100), enhancing antigen presentation and type I interferon signaling	RIG-I agonists synergized with checkpoint inhibitors to improve antigen cross-presentation in preclinical studies; ADU-S100 demonstrated innate and adaptive immune activation in early-phase trials	Overcoming variability in patient responses and optimizing delivery mechanisms for pathway-specific agonists	[188,189]
Tumor vaccines	Neoantigen-based mRNA vaccines deliver precise tumor antigen targeting, enhancing T cell priming and memory formation	BioNTech’s individualized mRNA vaccines showed clinical efficacy in enhancing antitumor immunity	Optimizing vaccine personalization and ensuring scalability for broader clinical applications	[190-192]
Oncolytic viruses (OVs)	Combines tumor lysis with immune activation; engineered OVs release cytokines (e.g., GM-CSF) to enhance antigen presentation and immune recruitment	T-VEC and CG0070 demonstrated clinical efficacy in enhancing immune responses and tumor control	Addressing resistance to viral infection and optimizing cytokine payloads for safety and efficacy	[193,194]
Cancer-specific adjuvants	Enhance APC activation and immune responses through TLR7/8 agonists, STING activators, and synthetic adjuvants, amplifying vaccine efficacy	Adjuvants combined with mRNA vaccines and OVs improved immune responses in checkpoint-refractory patients	Optimizing adjuvant combinations to minimize toxicity and ensure robust immune activation	[199-202]
Glycolysis and Lactate targeting	Reduces lactate-driven immunosuppression by inhibiting glycolytic enzymes (e.g., LDHA) and lactate transporters (e.g., MCT1)	MCT1 inhibitor AZD3965 demonstrated immune activation and metabolic correction in early-phase trials	Addressing potential off-target effects and optimizing combination with ICIs	[199-201]
Nutrient rebalancing	Restores T cell function under nutrient scarcity by blocking arginase/IDO pathways and modulating glucose allocation via GLUT inhibitors	IDO inhibitor epacadostat showed promising T cell restoration in preclinical studies	Balancing tumor and immune cell metabolism to minimize unintended effects on normal tissues	[202-204]
Mitochondrial function enhancement	Restores immune cell metabolism by boosting mitochondrial biogenesis (e.g., PGC-1α agonists) and reducing oxidative stress via ROS scavengers	PGC-1α agonists and mitochondrial autophagy enhancers showed improved T cell persistence and antitumor immunity in preclinical studies	Balancing oxidative stress mitigation with sustained immune activation to avoid overcompensation	[205-213]
Multi-modal immuno-metabolic reprogramming	Combines ICIs with metabolic agents to counteract lactate buildup (e.g., LDH/MCT1 inhibitors) and restore amino acid availability (e.g., IDO inhibitors)	LDH/MCT1 inhibitors and IDO blockade demonstrated enhanced CTL responses and metabolic reprogramming in preclinical studies	Developing dual-target therapies to optimize glycolysis inhibition and mitochondrial function without impairing normal tissues	[67,108,113,199-201,214]
Vascular remodeling with immune Co-activation	Combines angiogenesis inhibitors (e.g., VEGF/ANG2 blockers) with immune activators (e.g., STING agonists) to normalize vasculature and enhance T cell priming	Anti-VEGF + STING agonist combinations improved immune infiltration and antigen presentation in preclinical models	Balancing vascular normalization with sustained immune activation; optimizing multi-receptor antibody therapies for safety	[126-128,131,215-219]
Dynamic immune-network modulation	Disrupts immune exclusion via complement inhibitors (e.g., C3a/C5a blockers), NETosis inhibitors, and TLS-enhancing therapies (e.g., CXCL13 inducers)	Complement inhibitors and TLS enhancers demonstrated improved immune infiltration and pro-immunity niches in preclinical studies	Optimizing combinatory approaches to balance immune activation and mitigate potential off-target effects	[220-225]
Personalized therapeutic frameworks	Integrates cGAS-STING activators, DC vaccines, and oncolytic viruses with engineered cytokines to drive durable immune activation	cGAS-STING activators and DC vaccines showed enhanced immune responses and tumor control in clinical and preclinical studies	Balancing multi-pathway targeting to reduce toxicity and ensure adaptability for resistant tumors	[185,187,193-195]

VEGF: Vascular endothelial growth factor; ECM: extracellular matrix; CAFs: cancer-associated fibroblasts; CTL: cytotoxic T lymphocyte; MDSC: myeloid-derived suppressor cell; TAM: tumor-associated macrophage; CCR2: C-C chemokine receptor type 2; CCL2: C-C motif chemokine ligand 2; DCs: dendritic cells; CXCL9: C-X-C motif chemokine ligand 9; CXCL10: C-X-C motif chemokine ligand 10; CRISPR: clustered regularly interspaced short palindromic repeats; CCR4: C-C chemokine receptor type 4; CD25: interleukin-2 receptor alpha chain; CTLA-4: cytotoxic T-lymphocyte-associated protein 4; Treg: regulatory T cells; IDO: indoleamine 2,3-dioxygenase; CXCR2: C-X-C chemokine receptor type 2; CSF-1R: colony-stimulating factor-1 receptor; PI3Kγ: phosphoinositide 3-kinase gamma; TLR: Toll-like receptor; STING: stimulator of interferon genes; ICI: immune checkpoint inhibitor; TDE: tumor-derived exosomes; PD-L1: programmed death-ligand 1; TCR: T cell receptor; PD-1: programmed death-1; LAG-3: lymphocyte activation gene-3; CAR-T: chimeric antigen receptor T cell; ICOS: inducible T cell costimulator; 4-1BB: tumor necrosis factor receptor superfamily member 9; IL-7: Interleukin 7; IL-15: interleukin 15; PGC-1α: peroxisome proliferator-activated receptor gamma coactivator 1-alpha; NK cells: natural killer cells; AMPK: AMP-activated protein kinase; CAR-NK: chimeric antigen receptor natural killer cell; TME: tumor microenvironment; STING: stimulator of interferon genes; DC: dendritic cell; NSCLC: non-small cell lung cancer; cGAS: cyclic GMP-AMP synthase; RIG-I: retinoic acid-inducible gene I; FLT3L: FMS-LIKE TYROSINE KINase 3 ligand; GM-CSF: granulocyte-macrophage colony-stimulating factor; APC: antigen-presenting cell; T-VEC: talimogene laherparepvec; LDHA: lactate dehydrogenase A; MCT1: monocarboxylate transporter 1; GLUT: glucose transporter; LDH: lactate dehydrogenase; ANG2: angiopoietin-2; C3a: complement component 3a; C5a: complement component 5a; CXCL13: C-X-C motif chemokine ligand 13; NETosis: neutrophil extracellular trap formation; TLS: tertiary lymphoid structures.

### Enhancing immune infiltration

Effective immune infiltration is pivotal for overcoming immunotherapy resistance in NSCLC, yet this is often obstructed by structural, biochemical, and cellular barriers. Current advances target tumor vasculature normalization, ECM remodeling, and chemokine rebalancing to promote effector immune cell infiltration and activation.

#### Overcoming physical barriers

Dysfunctional tumor vasculature driven by VEGF overexpression leads to hypoxia, nutrient deprivation, and immune exclusion. VEGF also downregulates endothelial adhesion molecules essential for T cell trafficking^[85,86,125]^. New-generation VEGF inhibitors, including bispecific antibodies targeting VEGF and Ang2 (e.g., BI 836880), stabilize tumor vasculature, enhancing immune cell extravasation^[126-128]^. Moreover, combinations of VEGF inhibitors with ICIs, such as atezolizumab plus bevacizumab, have shown significant clinical benefit by improving oxygenation and effector T cell access^[18,20,129-131]^.

The ECM, largely shaped by CAFs, forms a fibrotic barrier that limits immune infiltration while fostering tumor progression^[132,133]^. Emerging ECM-targeting approaches, including antifibrotic agents (e.g., losartan)^[134,135]^ and ECM-degrading enzymes (e.g., PEGPH20 targeting hyaluronan)^[136,137]^, disrupt this barrier. Additionally, CAF reprogramming therapies leveraging retinoic acid receptor agonists or CXCR4 inhibitors promote a shift from pro-tumorigenic to immune-supportive fibroblasts, facilitating CTL migration^[138,139]^.

#### Chemokine rebalancing and immune cell trafficking

Chemokine dysregulation skews immune cell recruitment toward suppressive phenotypes. Tumors frequently secrete CCL2 and CXCL5 to attract MDSCs and TAMs while reducing CXCL9/10, thereby limiting CTL recruitment. Precision-targeted therapies aim to reverse these gradients: CCR2 inhibitors block CCL2-mediated MDSC recruitment, while DCs engineered to express CXCL9/10 restore CTL attraction^[140-144]^. Furthermore, gene-editing technologies such as CRISPR-Cas9 enable *in vivo* chemokine modulation, enhancing immune surveillance^[145,146]^.

These integrative strategies collectively reshape the tumor milieu, enabling deeper and more sustained immune infiltration, ultimately converting immune-excluded NSCLC into immune-responsive, treatment-sensitive tumors.

### Modulating immunosuppressive mechanisms

The immunosuppressive landscape of NSCLC is sustained by intricate networks involving Tregs, MDSCs, TAMs, and TDEs. Disrupting these suppressive pathways is pivotal for reshaping the TME into a more immune-permissive state.

#### Targeting tregs

Tregs mediate immune suppression by producing IL-10 and TGF-β while inhibiting effector T cells. Strategies target Treg recruitment and function using chemokine inhibitors, such as CCR4 antagonists^[147]^. Engineered monoclonal antibodies, including anti-CD25 agents with selective depletion properties, reduce peripheral and intratumoral Treg populations while sparing activated effector T cells^[148]^. Emerging candidates, such as anti-CTLA-4 variants with modified Fc domains, specifically deplete Tregs in the TME, enhancing antitumor immunity^[149]^.

#### Disrupting MDSCs

MDSCs suppress immune responses through arginine depletion, oxidative stress induction, and T cell inhibition via immune checkpoints such as PD-L1. Selective inhibitors of arginase (INCB001158) and IDO (epacadostat) are advancing in clinical trials^[150,151]^. CXCR2 inhibitors (e.g., SX-682) prevent MDSC recruitment and reverse immune exclusion^[152]^. Multi-omics profiling now supports real-time MDSC monitoring, enabling precision-targeted interventions that adjust based on the tumor’s evolving immune landscape^[119,120]^.

#### Reprogramming TAMs

TAMs exist along a phenotypic continuum from immunosuppressive M2-like to pro-inflammatory M1-like states. TAM reprogramming strategies include CSF-1R inhibitors (e.g., LY3022855), which reduce M2-TAM recruitment^[153]^. Small-molecule inhibitors targeting PI3Kγ further enhance M1 polarization, promoting phagocytic activity and boosting T cell priming^[154]^. Novel synthetic TLR agonists and cGAS-STING activators induce IFN-I production, reinforcing macrophage-driven antitumor responses while complementing checkpoint inhibitors^[155,156]^.

#### Neutralizing TDEs

TDEs act as vectors for immunosuppressive molecules, including PD-L1, IDO, and suppressive RNAs^[93,94]^. Innovative inhibitors targeting exosome biogenesis, such as neutral sphingomyelinase blockers (e.g., GW4869), disrupt TDE secretion^[157]^. Additionally, engineered nanobodies neutralizing exosomal PD-L1 have demonstrated the reversal of T cell exhaustion in preclinical models^[158]^. RNA-based exosome-editing therapies are emerging as a cutting-edge approach for altering the immunosuppressive cargo of TDEs^[159]^.

By integrating these precision-driven strategies, it becomes possible to dismantle the suppressive TME and restore antitumor immune activity. This comprehensive approach holds promise for advancing immunotherapy effectiveness and prolonging survival in patients with NSCLC.

### Activating dormant immune pathways

Reactivating dormant immune pathways in NSCLC is essential for reversing immunosuppression and enhancing therapeutic responses. Cutting-edge strategies aim to restore TCR signaling, promote memory T cell formation, reestablish NK cell cytotoxicity, enhance DCs antigen presentation, and leverage emerging immune activation pathways for sustained antitumor immunity.

#### Reinvigorating adaptive immunity

Enhancing TCR signaling Restoring TCR signaling is crucial for reversing T cell exhaustion in NSCLC. Dual checkpoint blockade, such as PD-1/LAG-3 inhibition, enhances effector function and T cell persistence^[160,161]^. TCR-engineered therapies combined with checkpoint inhibitors bypass TME-associated suppression, driving durable antitumor responses^[162]^.

Next-generation cellular therapies integrate advanced costimulatory domains like 4-1BB or ICOS and secrete IL-18 or PD-1-blocking agents to resist TME-induced inhibition^[163-165]^. Logic-gated chimeric antigen receptors (CAR)-T cells targeting dual antigens enhance specificity, minimizing off-tumor effects^[166,167]^. Gene-edited allogeneic CAR-T platforms offer scalable off-the-shelf solutions, expediting clinical deployment^[168]^.

Synthetic TCR platforms and TCR-mimic antibodies broaden therapeutic reach by targeting tumor antigens beyond classical TCR recognition. These innovations collectively advance TCR signaling modulation, enhancing adaptive immune activation for durable responses in immunotherapy-resistant NSCLC.

#### Promoting memory T Cell formation

Long-term immune surveillance hinges on the formation of memory T cells. IL-7 and IL-15 superagonists are pivotal for promoting memory differentiation and survival^[169,170]^. Recent studies on BCL-6 and TCF-1 activation have highlighted their roles in driving central memory T cell development^[140,171]^. Additionally, mitophagy regulation through PGC-1α enhances mitochondrial health, enabling memory T cell persistence^[172,173]^.

mRNA vaccine platforms, including personalized neoantigen vaccines, have emerged as promising tools for inducing potent memory T cell response^[174-176]^. These approaches have shown clinical efficacy by promoting long-term tumor control through persistent antigen stimulation.

#### Reactivating innate immunity

Restoring NK cell function Recent breakthroughs in metabolic reprogramming have overcome TME-imposed NK cell dysfunction. AMPK agonists and mTOR inhibitors have restored NK cell metabolism, enhancing cytotoxicity even under nutrient-deprived conditions^[177]^. Bispecific NK cell engagers, which link NK cells to tumor-specific antigens, such as AFM24, have demonstrated precise tumor targeting with reduced toxicity^[178]^.

Engineered NK cells expressing chimeric antigen receptors (CAR-NKs) have entered clinical trials, exhibiting superior persistence and resistance to TME suppression compared to traditional NK cell therapies^[179]^. Additionally, IL-12-based cytokine therapies such as NKTR-214 have redefined NK cell persistence in adoptive cell transfer protocols^[180]^.

#### Boosting antigen presentation via DC activation

DC activation is crucial for initiating adaptive immune responses. Novel TLR agonists (e.g., TLR7/8 and TLR9 agonists) enhance DC maturation, promoting effective antigen cross-presentation^[181-184]^. Additionally, STING agonists such as ADU-S100 amplify type I interferon production, enhancing DC recruitment and activation^[185]^.

Recent innovations include implantable biomaterial scaffolds delivering sustained antigen and adjuvant release, creating localized immune activation hubs^[186]^. Precision DC vaccines loaded with tumor-specific peptides have shown success in driving polyclonal T cell responses and enhancing response rates in checkpoint inhibitor-resistant NSCLC patients^[187]^.

#### Leveraging immune pathways

Emerging immune activation pathways, including RIG-I/MAVS and cGAS-STING, have gained prominence in reprogramming the suppressive TME. Selective RIG-I agonists, such as FLT3L analogs, have amplified antigen cross-presentation while synergizing with checkpoint inhibitors^[188]^. Meanwhile, cGAS-STING agonists like ADU-S100 have shown promise in activating both innate and adaptive immune responses by boosting type I interferon signaling^[189]^.

#### Immunotherapy enhancers

Tumor vaccines Neoantigen-based personalized vaccines, powered by next-generation mRNA technology, have enabled precise tumor antigen targeting. Recent breakthroughs include multi-epitope mRNA vaccines delivering sustained T cell priming and memory formation^[190,191]^. Platforms like BioNTech’s individualized cancer vaccine have demonstrated clinical efficacy in boosting antitumor immunity^[192]^.

Oncolytic viruses Oncolytic viruses (OVs), such as T-VEC and CG0070, have emerged as dual-action agents, combining direct tumor lysis with immune activation^[193]^. Recent advances include tumor-selective OVs armed with immune-stimulating cytokines, such as GM-CSF, enhancing antigen release and immune cell recruitment^[194]^.

Cancer-specific adjuvants Cancer-specific adjuvants, including TLR7/8 agonists, STING activators, and synthetic adjuvants, have amplified vaccine responses by enhancing APC activation^[195-197]^. Combined with mRNA vaccines and OVs, these adjuvants have elevated immune responses in checkpoint-refractory patients^[198]^.

Coordinating adaptive and innate immunity is key to overcoming tumor immune evasion and driving durable antitumor responses.

### Addressing metabolic dysregulation

Reprogramming the TME necessitates counteracting metabolic dysregulation, a major driver of immune suppression and therapeutic resistance. Tumor cells’ altered metabolism fuels immunosuppressive niches, starving immune cells and creating metabolic barriers. Advanced strategies target key metabolic axes to sustain antitumor immunity and restore TME balance.

#### Targeting tumor glycolysis and lactate accumulation

Tumor cells’ dependence on aerobic glycolysis produces lactate, acidifying the TME and impairing CTLs and NK cells. Targeting glycolytic regulators such as hexokinase-2 and lactate dehydrogenase A (LDHA) has demonstrated potential in reducing lactate-driven immunosuppression^[66,69,199]^. Inhibiting lactate transporters like MCT1 prevents extracellular lactate buildup, alleviating its inhibitory effects on TCR signaling and promoting antigen-specific responses^[113,200]^. Advanced inhibitors such as AZD3965 have entered clinical evaluation, offering metabolic correction and immune activation^[201]^.

#### Rebalancing nutrient availability

Metabolic competition for essential nutrients like glucose, amino acids, and lipids intensifies within the TME. Tumor-driven depletion of arginine and tryptophan through arginase and IDO pathways disrupts T cell proliferation. Dual blockade of these enzymes, using IDO inhibitors like epacadostat, has shown promise in preclinical models^[202]^. Recent efforts extend to modulating glucose allocation by targeting glucose transporter (GLUT) or promoting selective nutrient uptake in T cells through engineered cytokine delivery, sustaining effector functions under nutrient scarcity^[203,204]^.

#### Enhancing mitochondrial function

Mitochondrial fitness underpins immune cell survival, activation, and persistence. Tumor-induced oxidative stress damages mitochondrial integrity, suppressing T cell metabolism. Boosting mitochondrial biogenesis through PGC-1α agonists restores metabolic resilience, supporting cytotoxic immune responses^[173,205]^. Agents like ROS scavengers and mitochondrial autophagy enhancers such as urushiol A improve oxidative phosphorylation and mitochondrial autophagy separately, promoting memory T cell formation and durable antitumor immunity^[206-208]^.

Emerging metabolic interventions incorporate next-generation strategies such as epigenetic metabolic reprogramming^[209,210]^, engineered cytokines targeting TME metabolic hubs^[211,212]^, and multi-target small molecules that correct key metabolic imbalances^[205,213]^. Combining these strategies with ICIs or adoptive cell therapies holds transformative potential, establishing metabolically optimized TMEs conducive to durable antitumor responses^[202,205,209,213]^.

### Combining strategies for synergistic effects

Addressing the complexity of the TME requires multi-layered therapeutic combinations that target interconnected immunosuppressive, metabolic, and structural pathways. Integrating cutting-edge strategies promises to recalibrate the immune landscape and optimize therapeutic efficacy.

#### Multi-modal immuno-metabolic reprogramming

Emerging therapies integrate ICIs with metabolic rewiring agents targeting lactate metabolism, amino acid replenishment, and mitochondrial restoration^[67]^. LDH/MCT1 inhibitors counteract lactate accumulation, enhancing T cell function^[113,199,201]^, while IDO blockade restores tryptophan availability, reactivating CTL responses^[75,76,108]^. Novel dual-target therapies simultaneously inhibit glycolysis and fuel oxidative phosphorylation, supporting sustained effector cell activation in resistant tumors^[214]^.

#### Vascular remodeling with immune co-activation

Agents targeting VEGF, ANG2, and endothelin pathways simultaneously normalize vasculature, reduce hypoxia, and promote immune infiltration^[86,126-128,131]^. Combination strategies pairing anti-VEGF therapies with STING agonists, or engineered cytokines and Adoptive cell transfer amplify antigen presentation and T cell priming^[215-218]^. Novel multi-receptor antibodies linking angiogenesis inhibitors with immune activators are reshaping the therapeutic landscape, enhancing both vascular function and immune accessibility^[219]^.

#### Dynamic immune-network modulation

Disrupting immune exclusion mechanisms is advancing through complement-targeting agents, including C3a/C5a inhibitors, which block suppressive feedback loops^[220,221]^. NETosis inhibitors paired with ECM remodelers clear physical barriers while restoring effector cell infiltration^[222]^. TLS enhancers, driven by CXCL13-inducing therapies, reshape the immune landscape by establishing pro-immunity niches^[223-225]^. B cell-directed immunotherapies targeting suppressive signaling redefine B cell functions, supporting antitumor immunity^[225,226]^.

#### Next-generation adaptive combinations

Personalized therapeutic frameworks leveraging cGAS-STING activators, DC vaccines, and OVs are redefining immune activation^[185,187,193]^. These platforms synergize with engineered cytokines and T cell-stimulating agents, driving durable immune responses^[165,167,169,172,173,194,195]^. Emerging multi-pathway therapies targeting immune suppressors, metabolic stress sensors, and checkpoint regulators offer precision-driven, adaptive immune reprogramming for treatment-refractory tumors^[156,160,173]^.

These integrative strategies represent a paradigm shift in immune oncology, forging new pathways to overcome TME resistance and reshape therapeutic outcomes in NSCLC.

#### Safety considerations of combination strategies

Despite their promising therapeutic potential, integrative combination strategies may carry certain safety considerations. For example, combining ICIs with antiangiogenic agents could increase the risk of vascular-related adverse events such as hypertension and proteinuria^[227]^. Similarly, therapies targeting metabolic pathways to enhance immune responses might inadvertently alter systemic immune homeostasis, potentially heightening immune-related toxicities^[228]^. Additionally, combinations utilizing OVs and signaling modulators might trigger excessive inflammatory responses or cytokine release syndrome^[229]^. Therefore, careful clinical evaluation and rigorous monitoring remain crucial to effectively balancing efficacy and safety.

## EMERGING TECHNOLOGIES AND BIOMARKERS

Building on the integration of multi-targeted therapeutic strategies, the next frontier lies in harnessing cutting-edge technologies and precision biomarkers. Advances in molecular profiling, spatial omics, and computational modeling are redefining how tumor heterogeneity is understood and targeted. These innovations enable precise patient stratification, dynamic therapy adjustment, and real-time monitoring, paving the way for next-generation immunotherapy approaches^[21,22,26,118,120]^ [Figure 2 and Table 3].

**Table 3:** Emerging technologies and applications in TME research

**Technology**	**Advantages**	**Application example**	**Future prospects**	**References**
Spatial transcriptomics	Maps spatial and molecular heterogeneity of the TME	Defines NSCLC subtypes by analyzing TIM3-HAVCR2 and CD96-NECTIN1 interactions	Integrates spatial and molecular insights to refine patient stratification and guide immunotherapy	[118,119]
Spatial proteomics	Provides multiplexed protein profiling to identify immune-structural dynamics	Detects CD163^+^ macrophage and FOXP3^+^ Treg niches in high-grade NSCLC	Develops therapies targeting immunosuppressive niches and spatial immune organization	[230]
Multi-omics integration	Combines spatial and single-cell data to reveal metabolic and immune reprogramming	Identifies TAM-driven cholesterol efflux (via ABCA1) and iron export (via SLC40A1) as targets	Enhances precision therapies by linking metabolic remodeling with immune cell dynamics	[118]
Predictive spatial modeling	Simulates TME dynamics to forecast treatment outcomes	Predicts cytotoxic T cell spatial proximity as a prognostic marker in NSCLC	Implements real-time TME monitoring to support adaptive immunotherapy strategies	[230]
AI-powered spatial analytics	Unveils spatially resolved immune phenotypes (e.g., inflamed, immune-excluded) strongly linked to therapy response	AI-powered TIL analyzers predict ICI responsiveness and correlate TIL distribution with survival in NSCLC	Advances patient stratification and biomarker discovery to refine immunotherapy strategies	[233]
GeoMx DSP	Quantifies immune and stromal markers across distinct TME compartments	Identifies spatial correlations of immune markers (e.g., CD56⁺ NK cells) with survival and resistance mechanisms (e.g., VISTA)	Enables actionable biomarker-based pathways to overcome immune evasion and personalize treatments	[234]
Liquid biopsy and CyTOF	Provides non-invasive, high-dimensional monitoring of immune cell dynamics and therapeutic response	Characterizes exhausted CD8⁺ T cells (e.g., CD8⁺CD101^hi^TIM3⁺) and identifies baseline immune predictors for PD-1 therapy	Optimizes real-time treatment monitoring and adaptive immunotherapy adjustments	[235,236]
Nanoparticle-based delivery	Enables precise, localized delivery of immunomodulatory agents with minimal systemic toxicity	LNP-based RNA vaccines targeting KRAS mutations demonstrated durable immune responses in preclinical NSCLC models	Advances stimuli-responsive nanocarriers for targeted payload release in hypoxic and acidic TMEs	[237-243]
Smart tumor-specific carriers	Enhances selectivity and efficacy through ligand-functionalized and TME-triggered delivery mechanisms	EGFR-functionalized nanoparticles achieved localized delivery while sparing healthy tissues in NSCLC models	Develops multifunctional nanocarriers integrating diagnostic and therapeutic capabilities (theranostics)	[244-248]
ToC	Precise, real-time modeling of tumor-stroma interactions and immune dynamics; enables the assessment of personalized therapeutic responses	Evaluating patient-specific immune responses and resistance mechanisms to anti-PD-1 immunotherapy in NSCLC	Integration into clinical workflows for routine immunotherapy prediction and optimization	[231]
PDOs	High-throughput screening of drug sensitivity; recapitulates patient-specific genomic and phenotypic tumor	Personalized *ex vivo* testing to predict clinical response and guide individualized NSCLC treatment strategies	Expansion into routine clinical practice for tailored precision oncology	[232]

NSCLC: Non-small cell lung cancer; TIM3: T cell immunoglobulin and mucin domain-containing protein 3; HAVCR2: hepatitis A virus cellular receptor 2; CD96: T cell surface protein CD96; NECTIN1: nectin cell adhesion molecule 1; CD163: hemoglobin scavenger receptor; FOXP3: forkhead box P3; Treg: regulatory T cells; TAM: tumor-associated macrophage; ABCA1: ATP binding cassette subfamily A member 1; SLC40A1: solute carrier family 40 member 1; TME: tumor microenvironment; AI: artificial intelligence; TIL: tumor-infiltrating lymphocyte; ICI: immune checkpoint inhibitor; CD56: neural cell adhesion molecule 1 (NCAM1), expressed on NK and NKT cells; NK cells: natural killer cells; VISTA: V-domain immunoglobulin suppressor of T cell activation; CyTOF: cytometry by time-of-flight; CD8: cluster of differentiation 8; CD101: cluster of differentiation 101; PD-1: programmed death-1; LNP: lipid nanoparticle; KRAS: kirsten rat sarcoma viral oncogene homolog; EGFR: epidermal growth factor receptor; DSP: digital spatial profiling; PDOs: patient-derived organoids; ToC: tumor-on-chip.

### Decoding spatial and molecular heterogeneity

Understanding spatial and molecular heterogeneity in NSCLC has progressed from theoretical frameworks to actionable clinical insights, driven by advanced spatial profiling technologies. These technologies provide unprecedented clarity on how cellular composition and spatial organization within the TME influence therapeutic responses, reshaping NSCLC classification and treatment strategies^[58,118,119]^.

Emerging spatial omics technologies, including spatial transcriptomics and multi-omics integration platforms, have transcended conventional histopathology by enabling molecular profiling with spatial resolution^[36,119]^. Recent large-scale studies integrating single-cell RNA sequencing (scRNA-seq) and spatial transcriptomics have redefined NSCLC subtypes by unveiling precise immune-epithelial interactions. For example, LUAD-specific immune landscapes feature TIM3-HAVCR2 interactions, while lung squamous cell carcinoma (LUSC) exhibits CD96-NECTIN1-mediated cytotoxicity evasion. This study identifies TAMs, especially STAB1+ macrophages, as reprogrammed with enhanced cholesterol efflux via ABCA1 and iron export through SLC40A1, fostering immune suppression and tumor progression, positioning them as key players in metabolic remodeling and promising therapeutic targets^[118]^.

Beyond transcriptional profiling, spatial proteomics has emerged as a pivotal tool in dissecting protein-level heterogeneity. Technologies such as Imaging Mass Cytometry have enabled multiplexed spatial proteomics to map the NSCLC microenvironment with unprecedented resolution. This study uncovered key immune-structural dynamics, including the enrichment of CD163^+^ macrophage and FOXP3^+^ Treg interactions in high-grade tumors, creating immunosuppressive niches. In contrast, CD8^+^ T cells in low-grade tumors showed stronger spatial proximity to tumor cells, indicating enhanced cytotoxic potential and better prognostic implications. Additionally, B cell-enriched neighborhoods were associated with improved survival, underscoring the spatial organization of immune cells as a determinant of clinical outcomes. These findings advocate for therapeutic strategies tailored to the spatial architecture of TIME^[230]^.

Recent advancements in 3D *ex vivo* cancer models, particularly tumor-on-chip (ToC) and patient-derived organoids (PDOs), have further complemented spatial and molecular characterization efforts. The lung ToC system effectively recapitulates tumor-stroma interactions and immune cell dynamics within a physiologically relevant context, allowing precise, real-time assessment of patient-specific immune responses to anti-PD-1 therapies^[231]^. Similarly, patient-derived lung organoids accurately capture genomic and phenotypic heterogeneity of clinical tumors, serving as robust platforms for *ex vivo* drug sensitivity screening and personalized response predictions^[232]^. Despite current limitations, including variable efficiency in organoid establishment and challenges in sustaining long-term fidelity to original tumor phenotypes, these innovative 3D models represent critical advancements for personalized medicine.

Looking ahead, the fusion of spatial omics, predictive modeling, and high-throughput clinical screening holds the promise of transforming NSCLC treatment paradigms. By integrating these technologies into clinical workflows, researchers aim to achieve continuous, real-time monitoring of TME dynamics, enabling precision-guided immunotherapy.

### Leveraging novel biomarkers for precision therapy

The heterogeneous nature of NSCLC necessitates an adaptive biomarker-driven treatment strategy. The integration of spatial omics and artificial intelligence-driven analytics has catalyzed biomarker discovery with unprecedented precision. Recently, an AI-powered spatial tumor-infiltrating lymphocytes (TILs) analyzer has demonstrated that the spatial distribution of TILs reveals critical immune phenotypes - such as inflamed, immune-excluded, and immune-desert - strongly associated with ICI responsiveness, tumor mutational burden, and patient survival, highlighting the value of AI analytics in enhancing biomarker interpretability and therapeutic prediction^[233]^.

Offering a distinct perspective, the GeoMx Digital Spatial Profiling (DSP) System has significantly advanced the deciphering of spatial TME architecture in NSCLC. By quantifying immune markers across distinct compartments - tumor, immune cells, macrophages, and stroma - it has revealed spatial immune associations, such as enriched CD56^+^ NK/NKT cells and CD4^+^ T cells correlating positively with patient survival. Conversely, elevated VISTA and CD127 expression within tumor compartments indicate immune-resistance mechanisms, highlighting actionable pathways to overcome immune evasion. These findings underscore spatially resolved immune markers’ critical role in refining patient stratification and personalized immunotherapy^[234]^.

Furthermore, liquid biopsy has emerged as a cornerstone of real-time tumor monitoring, complemented by cytometry by time-of-flight (CyTOF), which enables high-dimensional, longitudinal immune-cell characterization from peripheral blood. CyTOF and multiplex cytokine profiling studies in NSCLC patients receiving anti-PD-1 therapy have shown that higher baseline frequencies of exhausted CD8^+^ T cells, particularly the CD8^+^CD101hiTIM3^+^ subset, correlate with poor therapeutic outcomes. In contrast, increased expression of cytotoxic markers, such as Granzyme B and T-bet, identifies potential responders. Baseline immune cell profiles - including classical monocytes, NK cells, and ICOS^+^ CD4^+^ T cells - also predict pembrolizumab efficacy, supporting non-invasive approaches for treatment monitoring and optimization^[235,236]^.

However, despite these technological advances, limitations remain. AI-powered analytics and spatial profiling techniques often require extensive high-quality datasets and complex computational infrastructures. Additionally, current platforms face constraints in the resolution, sensitivity, and interpretability of results, necessitating further technological refinement and validation. Addressing these challenges will accelerate the translation of multi-omics technologies, liquid biopsy innovations, and AI-driven predictive models into routine clinical practice, marking the next frontier in precision oncology for NSCLC.

### Precision durg delivery through nanotechnology

Recent advancements in nanoparticle-based delivery platforms have demonstrated remarkable potential in improving immunotherapy efficacy. Lipid nanoparticles (LNPs), as seen in mRNA vaccine technology, enable precise delivery of tumor-specific antigens, cytokines, and siRNAs targeting key immunosuppressive genes. Preclinical and early clinical studies have reported durable immune responses with LNP-based RNA vaccines targeting KRAS mutations in NSCLC, highlighting their translational potential^[237,238]^. Similarly, polymeric nanoparticles engineered for controlled release of immunomodulatory agents, such as PD-L1 inhibitors and STING agonists, have demonstrated superior therapeutic outcomes with minimal systemic toxicity^[239-241]^. Notably, hypoxia-responsive polymeric carriers incorporating oxygen boosters have successfully enhanced T cell infiltration in preclinical NSCLC models, offering a novel approach to overcoming hypoxic TME barriers^[242,243]^.

Tumor-specific nanocarriers that leverage ligand-functionalization and stimuli-responsive mechanisms provide enhanced selectivity and therapeutic efficacy. Nanoparticles functionalized with EGFR antibodies or folate receptor ligands have shown promising results by achieving localized drug delivery while sparing healthy tissues^[244,245]^. In parallel, TME-triggered carriers that respond to acidic pH, enzymatic activity, or oxidative stress ensure precise payload release at the tumor site^[246-248]^. Such smart delivery platforms are rapidly advancing toward clinical application, driven by their ability to amplify therapeutic effects while minimizing off-target toxicity.

## CONCLUSION

Advancing immunotherapy in NSCLC requires overcoming the intricate web of TME-driven resistance. This review has highlighted pivotal mechanisms such as immune suppression, metabolic reprogramming, and spatial heterogeneity that hinder therapeutic efficacy. Despite significant progress, persistent challenges remain in understanding dynamic TME remodeling and its evolving interplay with emerging therapies.

Future research must focus on leveraging spatial multi-omics, real-time biomarker monitoring, and precision delivery systems to enhance treatment specificity and adaptability. Single-cell technologies integrated with spatial proteomics and AI-powered predictive models hold the potential to uncover novel therapeutic targets while enabling patient-specific therapeutic designs. These innovations will likely redefine how we interpret TME dynamics and guide adaptive therapy regimens. Clinical translation will benefit from next-generation combination therapies, including ICIs paired with metabolic modulators, TME-targeting agents, and personalized cancer vaccines. Expanding biomarker-driven patient stratification and optimizing treatment timing through longitudinal molecular profiling will further improve therapeutic outcomes. Achieving sustainable progress in NSCLC immunotherapy will require multi-disciplinary collaboration spanning oncology, bioinformatics, immunology, and materials science.

Looking ahead, the integration of nanotechnology, AI-driven analytics, and immunotherapy offers an unprecedented opportunity to overcome resistance mechanisms and reshape the treatment landscape of NSCLC. As research converges toward personalized and adaptive immunotherapy, the path toward long-term survival and even potential cures becomes more tangible, driving a hopeful future in the fight against lung cancer.

## DECLARATIONS

### Copyright

© The Author(s) 2025.
